# Enhanced Microstructural and Mechanical Properties of Mig Welded Al 7075 Alloy Under Longitudinal Vibrations

**DOI:** 10.3390/ma18184281

**Published:** 2025-09-12

**Authors:** Teodor Machedon-Pisu, Mihai Machedon-Pisu

**Affiliations:** 1 Department of Materials Engineering and Welding, Transilvania University of Brașov, B-dul Eroilor nr. 29, 500036 Brașov, Romania; tmache@unitbv.ro; 2 Department of Electronics and Computers, Transilvania University of Brașov, B-dul Eroilor nr. 29, 500036 Brașov, Romania

**Keywords:** mechanical properties, microstructural characteristics, Al 7075 alloy, metal inert gas (MIG) welding, longitudinal vibrations, heat-affected zone (HAZ)

## Abstract

In many areas such as the automotive, aircraft, and building industries, the high-strength aluminum alloy Al 7075 is frequently used due to its appropriate properties as a lightweight structural material. However, due to modest weldability, it is challenging to obtain high-quality welds with suitable mechanical properties, as cracks are generated while welding. Moreover, in order to avoid post-welding heat treatments and the use of complex welding equipment, in this paper the Al 7075 alloy is welded with MIG under longitudinal vibrations by using the Al 4043 alloy as filler material. As a consequence of strengthening the HAZ through precipitation, the mechanical and structural properties of the welded joints can be improved. These are investigated both under longitudinal forced vibrations at 50 Hz and without such vibrations. The results reveal improvements in terms of reducing the risk of hot cracking, obtaining a band structure free of porosity of the welds, improving the hardness of the welds under vibrations by 8.7% to 12.5%, and improving the tensile strength of the plates welded under vibrations by 12 to 15.5% in comparison to no vibrations. In relation to other welding procedures, the proposed procedure is more cost-effective and the weld quality is improved during the welding process.

## 1. Introduction

The use of aluminum is closely related to the cumulative benefits that it offers in many industrial areas such as aerospace, the automotive industry, construction, machinery, sensors, and the military [1,2,3,4,5,6,7,8,9,10]. These benefits include reduced cost, low density, high thermal conductivity and anticorrosion [3,6,7,8,11]. High-strength aluminum alloys belonging to the 7xxx series are widely used as lightweight structural materials presenting excellent properties related to specific strength, toughness, stiffness, hardness, machining performance, and corrosion resistance [2,3,4,6,7,8,9,10,12].

The aluminum 7xxx series is a heat-treatable group of alloys that incorporate both weldable and non-weldable chemical compositions [2,5,12,13,14]. In order to meet the corresponding internal standards and norms, it must be mentioned that high-quality welds of these aluminum alloys are a key element in manufacturing automotive and aircraft structure components, as noticed in a few studies [4,5]. Improvements in this field without heat treatments or complex welding equipment [11,15,16] are important due to the need to scale down production costs, as noticed in [10]. This also leads to a higher efficiency in time spent, as well as offering capabilities such as pollution control and energy savings, as depicted in [12,14]. Other relevant aspects related to the above-mentioned issues refer to improvements in fatigue response [11,15,17,18,19], machinability [20], strengthening [17,21,22,23,24,25,26,27,28], corrosion resistance [23,28], productivity [29], wear resistance [29], and stability [30].

However, the advantages that certain aluminum alloys from the 7xxx series bring as a consequence of their physical, chemical and mechanical properties can be compromised when trying to use them in technical environments for which they have not been designed. This is especially the case with the AlZn5.5MgCu alloy, otherwise known as the 7075 series or the Al 7075 alloy, which is a heat-treatable alloy with impressive tensile and yield strength [4,6,8,12] but with poor weldability [5,12,13]. The most widely used technologies for welding Al 7075 alloys are tungsten inert gas (TIG) [13,23,25] and metal inert gas (MIG) welding [5,11], plasma arc welding [21], laser welding [6,12,17], and friction stir welding (FSW) [14,15,16,18,24,26]. Although FSW has gained a lot of interest recently, most studies do not consider the negative impact on production and labor costs, the requirements which result from the use of expensive welding devices [14,18,26], or post-welding treatments [15,16,24]. Such expenses, among others such as consumables and maintenance costs, are also relevant in the case of plasma arc welding and laser welding [6]. Both TIG and MIG are better suited when it comes to lower costs and higher productivity [23,25], with MIG being slightly better in productivity [5].

A large number of research studies focus on the analysis of structural and mechanical characteristics of Al 7075 welded joints in terms of microstructure [17,21,24,25,27,31,32,33,34,35,36,37,38,39,40,41], hardness [17,21,26,33,34,36,42,43,44,45], grain structure [22,26,40,44,45,46,47], and mechanical properties [16,17,21,25,32,34,35,36,38,39,40,42,47,48,49,50], as well as cracks [5,18,37,51,52], residual stress [53], and elastic-plastic strain [54]. Also, non-conventional measurement methods can be applied in this regard, such as in [55].

It can be noticed that in many of these studies, such as [6,11,12,14,15,16,17,18,21,22,23,24,25,27,31,36,38,39,42,44,47,50,54], post-processing treatments are applied in order to improve the structural and mechanical characteristics of Al 7075 welded joints. Out of the mentioned studies, only [36] does not treat any aspect regarding the generation of cracks.

At the same time, it must be mentioned in this regard that the majority of these studies fail to emphasize the technical aspects regarding mechanical vibrations that can have a significant influence on microstructure and, implicitly, on the evolution of cracks. Yet a few studies mention these aspects, such as [23,29,30,39,45,54]. Vibrations are shown to have an influence on the grain structure in [23,54]. The effects of ultrasonic vibrations on mechanical properties are mentioned in [39,45].

In terms of microstructure analysis, which is related to the presence of cracks in aluminum 7xxx series welded joints, it is well-known that alloys like Al, Zn, and Mg have a higher tolerance to hot cracking and show better welding performances, in comparison to Al, Zn, Mg, and Cu alloys [56,57]. In Al, Zn, and Mg alloys, the quantity of magnesium increases the sensitivity to cracking, a reaction that can be reduced by adding zirconium, in order to obtain a more refined grain size structure, thus improving the mechanical properties of the weld, as shown in [2,58].

In relation to MIG welding feasibility, the Al 7075 alloy can be affected by stress corrosion cracking, which is why it is not usually welded, as the propensity to crack while welding occurs due to the compound of Cu with Zn and Mg in the alloy, thus intensifying the solidification stress and leading to grain boundaries cracking, as discussed in [59]. As a difference, an alloy such as Al 7003, which has a small amount of copper (between 0.2% and 0.4%), is specially designed for high-strength welding [60]. Although the Al 7075 alloy is considered unsuitable for welding because of its increased probability of stress-induced corrosion [8], its physical characteristics are very interesting despite its low density of 2.81 g/cm^3^ [11].

It is worth mentioning that one important property of all 7xxx alloys is the capacity of the heat-affected zone (HAZ) to become strengthened through precipitation after welding through post-welding treatments, as discussed in numerous studies [4,9,10,11,12,13,14,15,18,19,21,24,25,27,32,34,36,37,38,41,42,44,46,47,48,49,50,58,59]. In many of these studies, the authors manage to obtain a decrease in the size of grains by reducing the HAZ.

As mentioned previously in [23,29,30,39,45,54], the presence of mechanical vibrations at various frequencies can also affect the grain structure. By manipulating the frequency, there is an obvious difference in structure with new sets of different characteristics. The differentiation of property efficiency can be established as efficiently as possible by starting from the classic welding procedure and moving toward a level of influence in the vibration frequency of the weld, which can highlight the reactions that occur. In this regard, a reduction in hot cracking in the susceptible area of an Al 7075 weld with TIG and MIG was observed by authors in [61] by subjecting it to mechanical vibrations at frequencies of 1025 and 2050 Hz, which has also led to a superior refinement of the structure in comparison to welding at higher and lower frequencies. Ultrasonic vibrations (beyond 20 kHz) have been applied during TIG welding of Al 5A06, Al 5A02 and Al 4047 in [62], revealing an increase in joint strength as well as increased microhardness in both the HAZ and the weld, as a consequence of grain refinement. Another study [63] discusses ultrasonic vibrations during welding of aluminum alloys from 5xxx and 6xxx series in terms of bonding for achieving proper welding, also addressing the major limitation of ultrasonic welding regarding its power delivery capability.

By carrying out MIG welding with longitudinal vibrations at lower frequencies, such as 52 Hz in [64] for an Al 6082 alloy, it was shown that weld solidification can have a major influence on the quality of the joining material, leading to finer and more uniform grains and better hardness in both the HAZ and the weld. Another study that applied vibrations during MIG welding [65] but at a lower frequency (10 Hz for an Al 6061 alloy) has shown that the grain growth of HAZ is slowed down and the influence of heat input on the base metal is decreased, also increasing the hardness in the base metal and the HAZ.

As shown in [66], coarse-grained structures (dendrites) appear in welds without forced vibrations. It can be interpreted that vibrations can improve the structural and mechanical properties of welded joints, as presented previously in [23,39,54,61,62,63,64,65,66]. But, in order to create beneficial changes in the metallographic structure of the welded joints with the contribution of longitudinal vibration, it is imperative to have permanent control over the vibrational course and its direction, and also to control the thermal influences. Various studies focused on this area describe various ways of introducing forced vibrations into the weld bed, such as electric arc vibrations [67,68] or introducing waveguide forced vibrations [69]. Mechanical vibrations can lead to beneficial structural changes and improve mechanical features of the finished material. The authors highlight the fact that full control is present during welding, without requiring any post-welding treatments.

In this study the authors apply forced longitudinal mechanical vibrations to an Al 7075 alloy welded with MIG at frequencies around 50 Hz. As a consequence, the mechanical and structural properties of the welded joints are investigated within.

## 2. Materials and Methods

The study has the comparative purpose to identify the mechanical, physical and chemical properties of the welds obtained with and without vibrations. In this regard, the experimental procedure consists of the mechanized welding on a ceramic support of two sets of Al 7075 (AlZn5.5MgCu, Alro S.A., Slatina, Romania) plates with dimensions of 300 mm × 150 mm × 10 mm. MIG welding equipment [70] is placed on a vibrating surface with a mechanized linear welding torch manipulator used in straight line welding. The MIG equipment (Air Liquide, Paris, France) was set to a 4-tact welding setup so that the beginning of the electric arc and the start of the welding torch travel were synchronized accordingly. For both procedures of welding, with and without vibrations, the welding torch was fixed on the arm of the mechanized equipment and positioned vertically so it could perform flat welding.

The Al 7075 experimental specimens that were to be welded were clamped on a metallic platform that had springs at every corner. To the vibrating metallic plate, an electrodynamic exciter was attached, in order to propagate vibrations in to the platform. The vibrating platform oscillates horizontally in the same direction as the weld is formed with a frequency-dependent acceleration. To power and control the electrodynamic vibration system, the authors used a power amplifier, a low-frequency generator with a frequency control system, a multimeter and frequency measurement system, in order to ensure the right frequency. The induced longitudinal vibration parameter used in the analysis has a frequency of 50 Hz. In accordance with recommendations of specialized companies and validations from industrial experience, the values for the experimental and technical welding parameters are determined as presented in Table 1, where I is the welding current, U is the supply voltage, f is the welding frequency, S is the welding speed, Sfw is the filler material speed, and Q Argon is the Argon gas flow.

The chemical composition of the Al 7075 alloy is given in Table 2.

For the welding procedure the authors used the Al 4043 alloy (AlSi5, Air Liquide, Paris, France) as filler material. Its chemical composition is depicted in Table 3.

Al 4043, or AlSi5, is a 4xxx series aluminum silicon-based alloy that contains a high level of silicon, as depicted in Table 3. By alloying aluminum with silicon, the alloy is given a set of properties with specific characteristics, such as reduced melting point [12,59] and excellent weldability [12]. Moreover, it improves the fluidity of the molten metal [12,32]. This is the reason why this alloy is especially used as filler material, as presented in [32,59,64,72,73,74]. Silicon, independently of aluminum, does not respond well to high temperatures, as noticed in [32], but some of these silicon alloys have been produced with additions of magnesium and copper, which enhance the ability to respond favorably to high-temperature welding, as shown in [12,32]. Silicon-based filler metals such as Al 4043 alloy (AlSi5) are not suitable for welding alloys like Al 7075 (AlZn5.5MgCu), since the Si surplus introduced by the filler wire leads to the formation of excessive quantities of fragile Mg_2_Si grains in the weld, as noticed in [12,32,59]. Most of the studies mentioned previously, such as [4,9,10,11,12,13,14,15,18,19,21,24,25,27,32,34,36,37,38,41,42,44,46,47,48,49,50,58,59], apply heat treatments after welding in order to strengthen the alloy by the precipitation of Mg_2_Si, as highlighted in [32]. However, in this study the strengthening occurs during welding. In this regard, the strength of a weld is established based on its size and by the shear strength of the filler metal used. In aluminum welding it is possible to select one of several different filler alloys to weld a particular aluminum alloy, with the most suited candidates as filler material being Al 4047 [75], 4643 [76], 5183 [77,78], 5356 [32,73], and 5554 [79], besides Al 4043. Based on the characteristics of the weld standpoint, it should be mentioned that in selecting the correct filler metal, the shear strength is an important factor, as depicted in [80,81]. From the comparison in Table 4, it can be noticed that the Al 4043 alloy has a lower level of shear strength, especially when compared to 5xxx series [82].

The superior mechanical resistance characteristic of the Al 7075 alloy and the capability of the HAZ to become strengthened through precipitation, in combination with the high fluidity of the Al filler material 4043, create a suitable environment for the alloy elements in the Al 7075 base material to precipitate in the HAZ of a weld formed with a lower-melting-point alloy that can withstand high temperatures due to additions of Mg and Cu. By introducing forced longitudinal vibrations in to the weld bed, the authors predict that the risk of hot cracking can be reduced, as discussed in [59], and a lower level of hydrogen inclusions in a higher-density grain microstructure (fragile Mg_2_Si grains) can be obtained, which is desired in [12,32,59].

In this regard, from each welded plate on a flat ceramic surface, the following samples were taken for laboratory mechanical tests and macroscopic and microscopic examinations:

- Two specimens of each welding procedure for macroscopic and microscopic examination;
- Two specimens of each welding process for the Vickers HV 100 microhardness test;
- Three specimens of each welding procedure, cut with water jets, according to ISO 4136: 2013 [83] dimensions for a transverse tensile test;
- Two specimens of each welding procedure for SEM coupled with EDX analysis (TESCAN, Brno, Czech Republic).

## 3. Results

**Figure 1:**
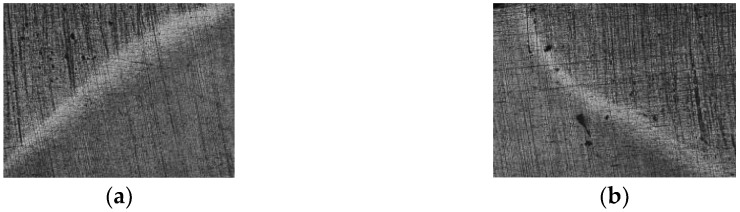
Macroscopic image of the test specimens welded: (**a**) without vibrations; (**b**) with vibrations; magnification: 10×.

Figure 1 represents photographs of MIG fusion weld structures of the test samples without and with forced longitudinal vibrations.

It can be observed in Figure 1a that the MIG welding procedure without vibrations consists in a considerable growth in the number of gaseous pores.

### 3.1. Chemical Composition Concentrations and Elemental Mapping Analysis

**Figure 2:**
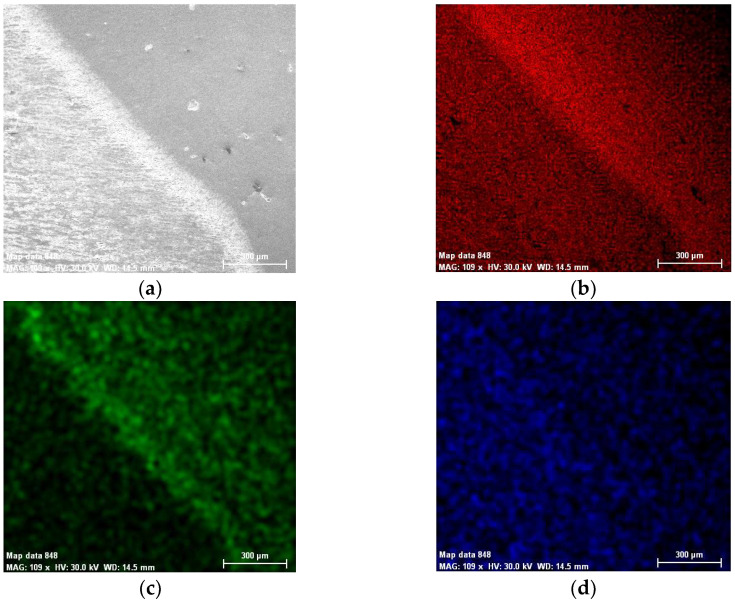

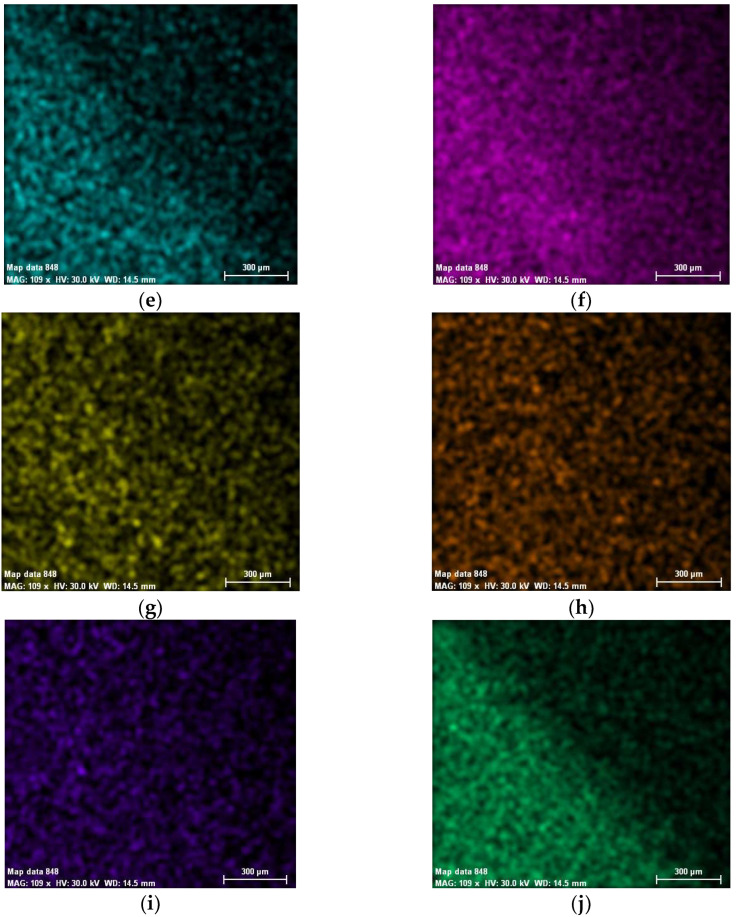
Chemical composition concentrations of the cross-section of the weld produced by MIG welding without longitudinal vibrations: (**a**) SEM image; (**b**) Al; (**c**) Si; (**d**) Cr; (**e**) Cu; (**f**) Mg; (**g**) Fe; (**h**) Ti; (**i**) Zn; (**j**) Mn.

**Figure 3:**
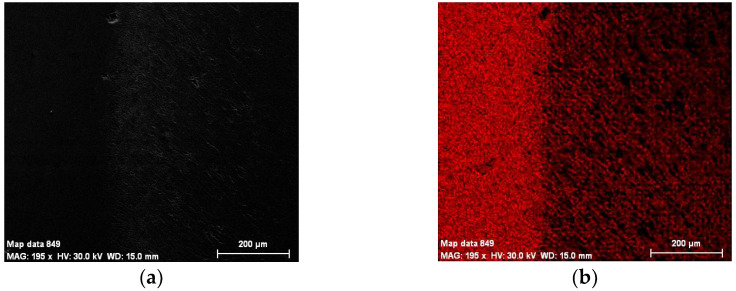

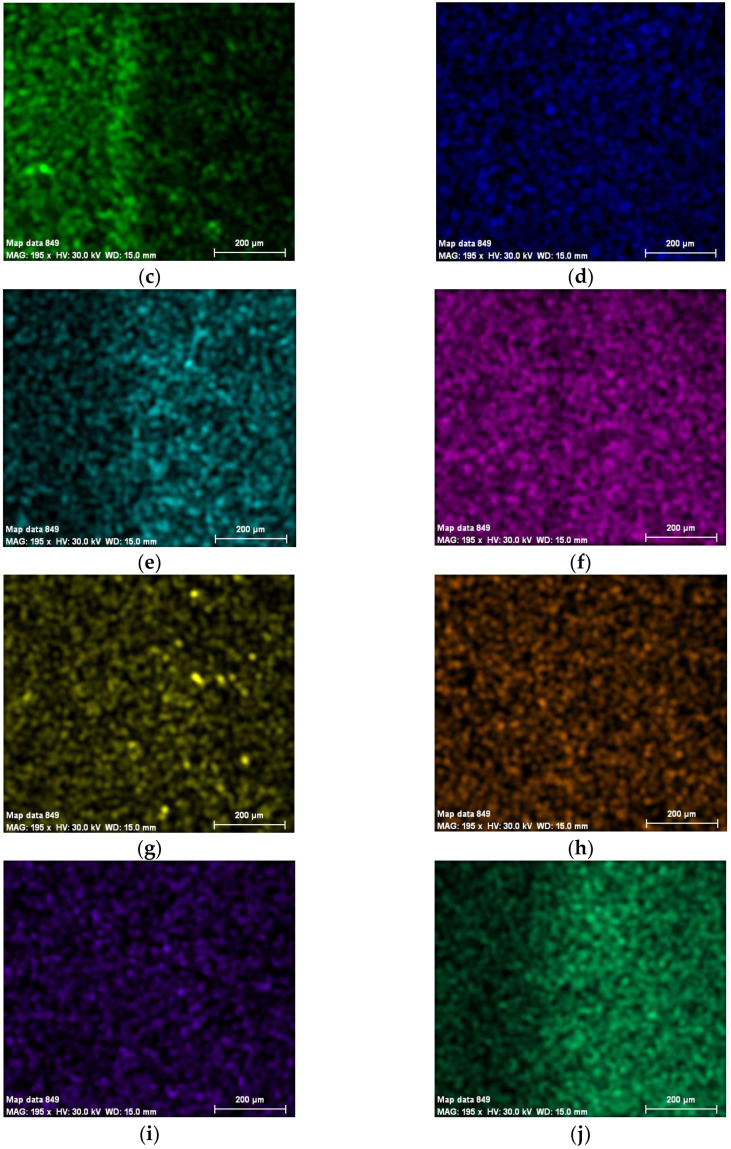
Chemical composition concentrations of the cross-section of the weld produced by MIG welding with longitudinal vibrations: (**a**) SEM image; (**b**) Al; (**c**) Si; (**d**) Cr; (**e**) Cu; (**f**) Mg; (**g**) Fe; (**h**) Ti; (**i**) Zn; (**j**) Mn.

The chemical composition concentrations of the cross-section of the weld and base material produced by MIG welding in terms of metallic elements in the two cases, without and with vibrations, are presented in Figure 2 and Figure 3, together with a Scanning Electron Microscopy (SEM) image.

**Figure 4:**
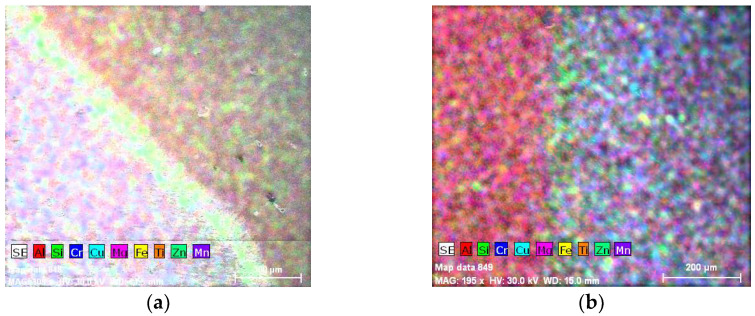
Composite elemental map in the weld produced by MIG welding: (**a**) without vibrations; (**b**) with vibrations.

In MIG welding, the subsequent stages of solidification of the molten pool are visible in Figure 4, being represented by the lighter-color lines arranged circumferentially at every few μm beginning from the fusion line up to the weld face boundary.

**Figure 5:**
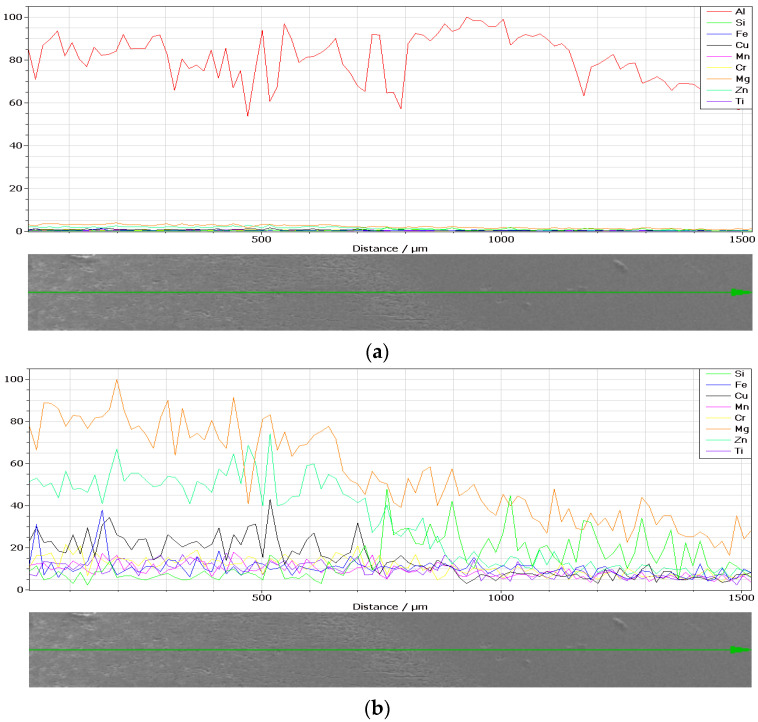
Chemical composition concentrations (in number of counts) in the weld produced by MIG welding without longitudinal vibrations of (**a**) metallic elements and (**b**) metallic elements except Al.

**Figure 6:**
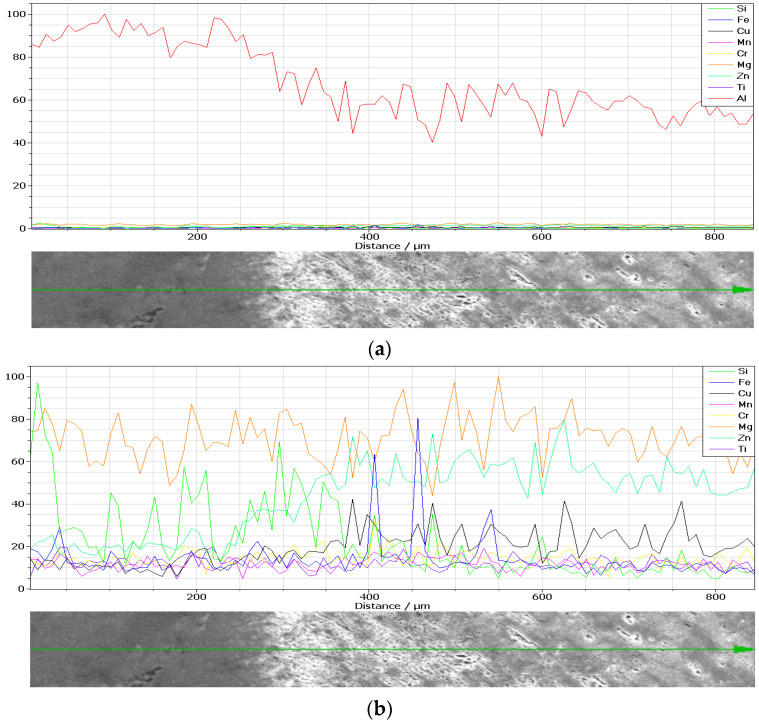
Chemical composition concentrations (in number of counts) in the weld produced by MIG welding with longitudinal vibrations of (**a**) metallic elements and (**b**) metallic elements except Al.

The metallic compositions obtained in the weld in the two cases, without and with longitudinal vibrations, are depicted according to Figure 5 and Figure 6.

By normalizing the metallic chemical concentrations depicted in Figure 5 and Figure 6 in number of counts, the experimental concentrations are determined in Table 5 and Table 6 in percent.

### 3.2. Scanning Electron Microscopy of the HAZ and Cross-Section of the Welds

**Figure 7:**
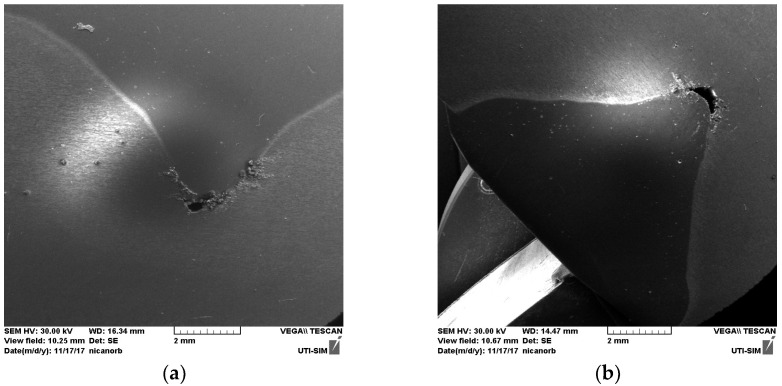
Microstructure in the HAZ of the MIG weld: (**a**) without longitudinal vibrations; (**b**) with longitudinal vibrations; magnification 20×.

**Figure 8:**
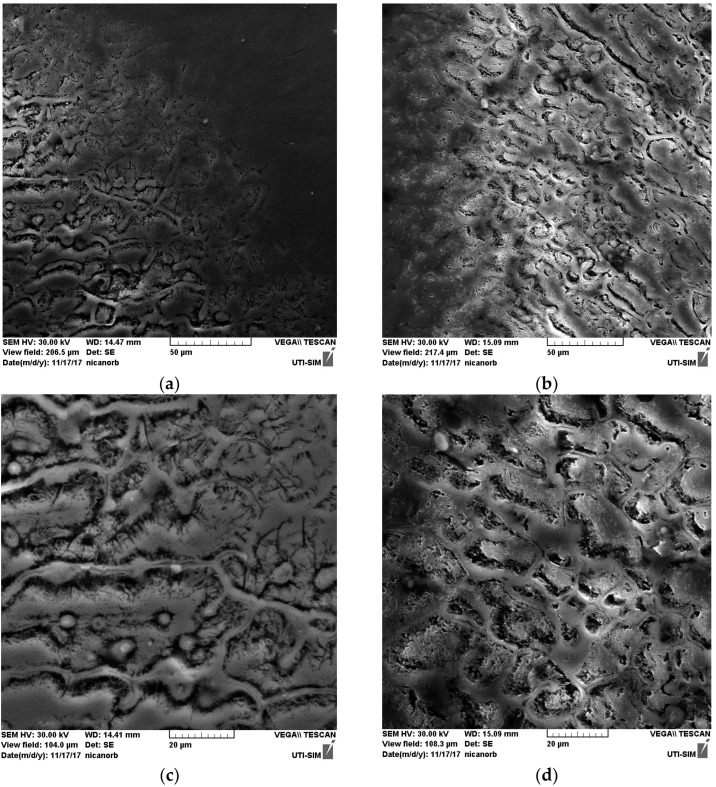

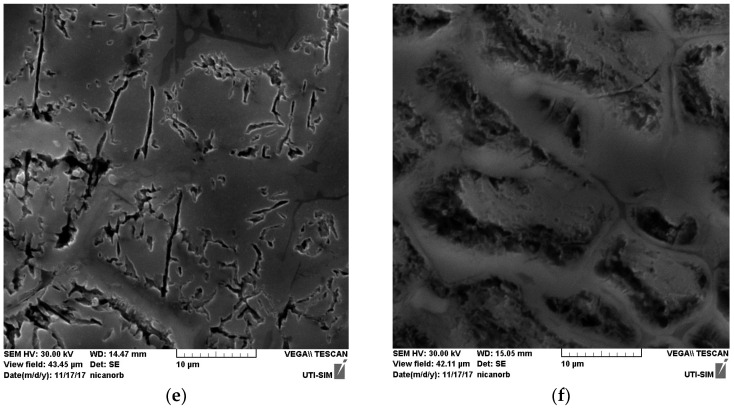
Microstructure in the HAZ of the MIG weld, magnification: (**a**) 1000×, without vibrations; (**b**) 1000×, with vibrations; (**c**) 2000×, without vibrations; (**d**) 2000×, with vibrations; (**e**) 5000×, without vibrations; (**f**) 5000×, with vibrations.

**Figure 9:**
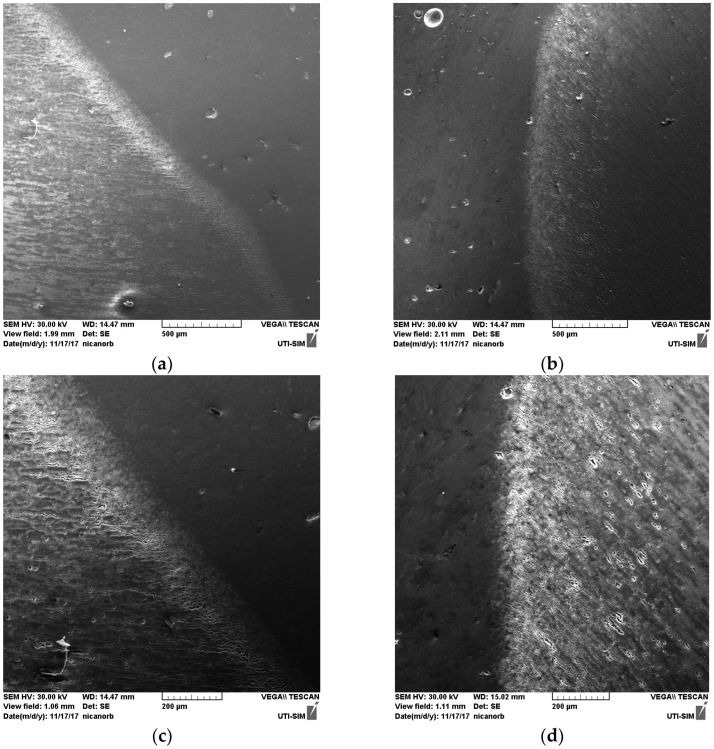

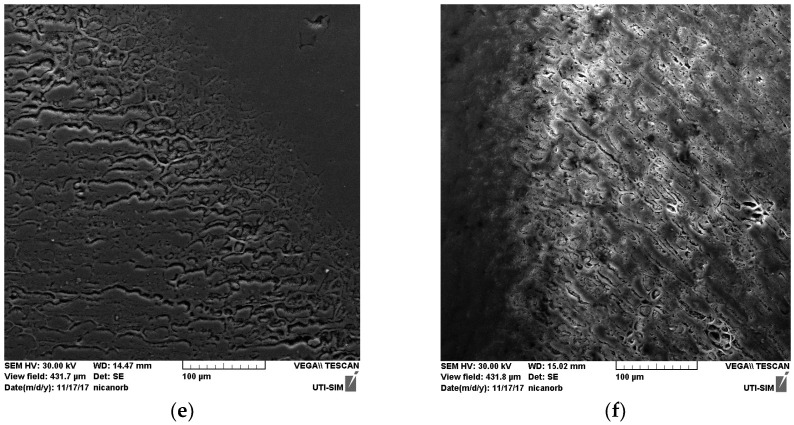
Microstructure in the cross-section of the MIG weld, magnification: (**a**) 100×, without vibrations; (**b**) 100×, with vibrations; (**c**) 200×, without vibrations; (**d**) 200×, with vibrations; (**e**) 500×, without vibrations; (**f**) 500×, with vibrations.

The cross-sections of the HAZ and weld’s morphology are examined in detail by using SEM in Figure 7, Figure 8 and Figure 9.

It can be seen that within the bands, the concentrations of silicon and copper are increased, especially in the MIG welds obtained with the participation of vibrations. At the nodes of the vibration wave, the structure appeared to be more advantageous than at its maximum. It can be seen in Figure 4 that Si and Cu have precipitated from the solid solution. This effect occurs in both displacements.

### 3.3. Hardness Determination of Specimens

The hardness of the aluminum alloys can be influenced by variables which include microstructural elements, chemical composition and strength. Vickers HV microhardness (100 g) was established using microhardness equipment (FUTURE-TECH, Kawasaki, Japan) which allows micrometer measurements using a force of up to 1000 g. The software analyses the structure according to the change in hardness and exerts a force of 100 g for 10 s. For each sample, three measurements were performed in the heat-affected layer and in the base material. The results presented in the tables below represent the average of the measurements.

#### 3.3.1. Welded Specimens Without Longitudinal Vibrations

Table 7 and Table 8 present the microhardness determinations for the welded specimens without longitudinal vibrations:

#### 3.3.2. Welded Specimens with Forced Longitudinal Vibrations

Table 9 and Table 10 present the microhardness determinations for the welded specimens with forced longitudinal vibrations:

### 3.4. Transverse Tensile Test of Welds

**Figure 10:**
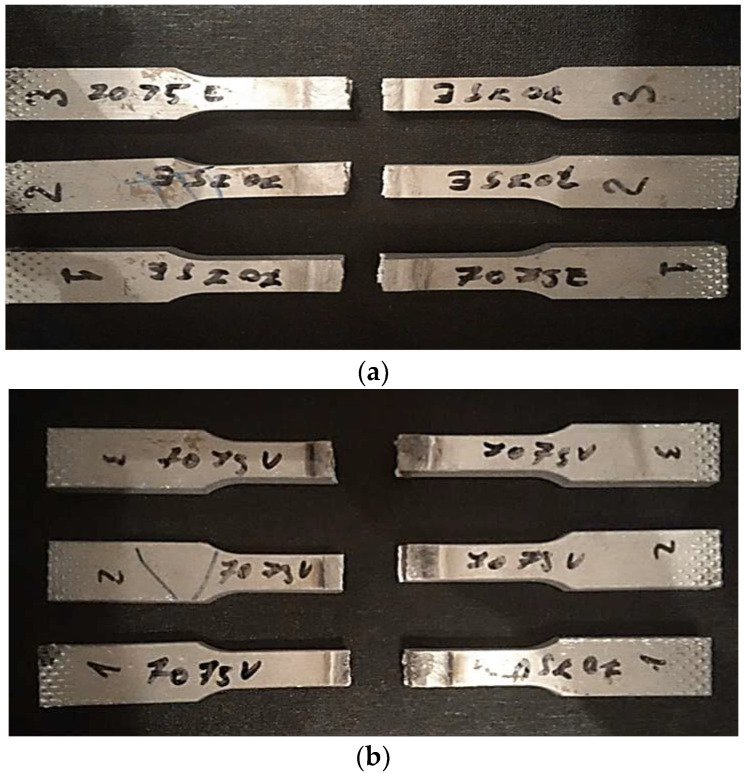
Test pieces: (**a**) welded on a static surface; (**b**) welded with longitudinal vibration.

The tensile laboratory tests were performed at ambient temperature (24 °C) and in accordance with EN ISO 4136: 2013 [83]. In Figure 10 the specimens are shown after the tensile test was performed. In Figure 10a the test pieces presented are welded on a static surface. In Figure 10b the test pieces presented are welded with longitudinal vibration. As the images show, in both welding procedures the specimens were torn as expected, inside the structure of the weld, near the HAZ. This result can have multiple causes, but in this particular condition, the authors have to take into consideration the metallographic changes that occur in the molten pool based on two alloys with a completely different behavior on welding, which are set to highlight the level of impact imposed by two opposite welding conditions.

**Figure 11:**
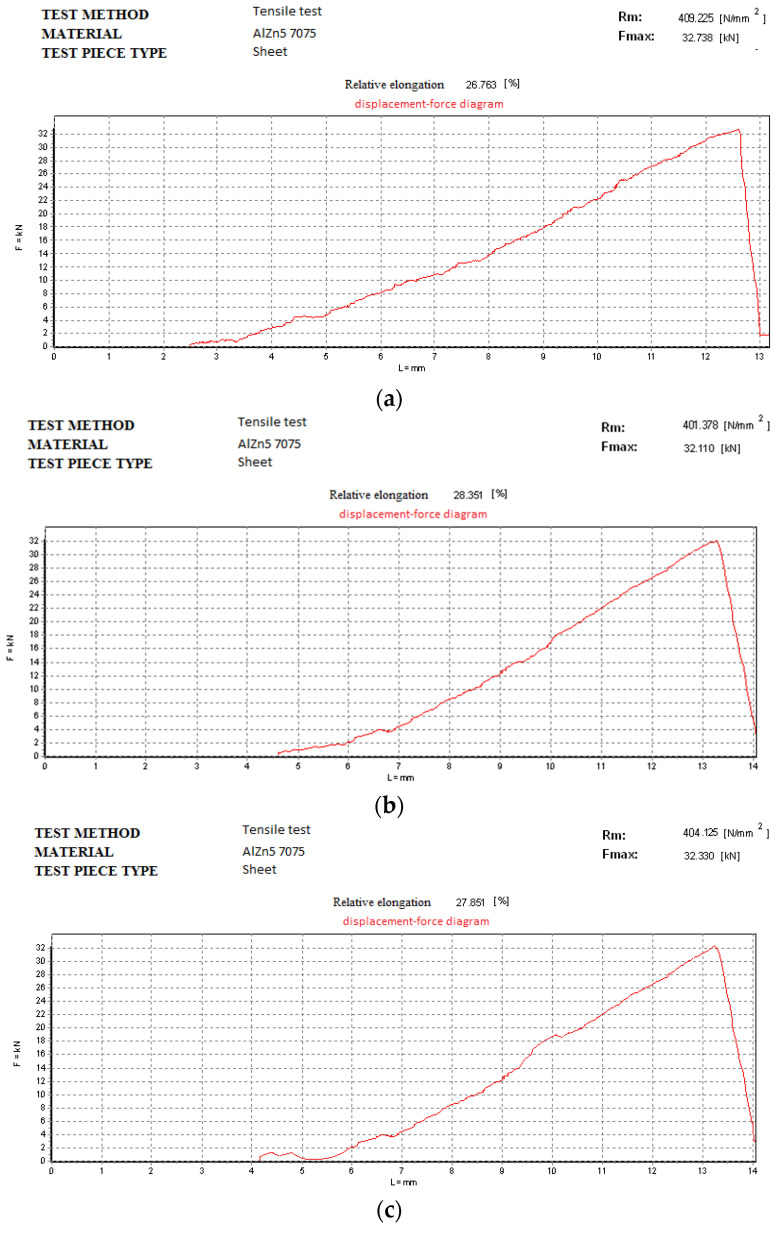
Tensile test for the specimens welded without vibrations: (**a**) test piece 1; (**b**) test piece 2; (**c**) test piece 3 from Figure 10a.

In accordance with EN 485-2: 2016 [84], the tensile strength of the AlZn5.5MgCu alloy is 540 MPa compared to 180 MPa for AlSi5, factors which determine that the yield point will be in the weld not in the base material. The results for the tensile tests on both procedures are presented in the figures below. Figure 11 depicts the graphs for the test specimens welded without vibrations.

**Figure 12:**
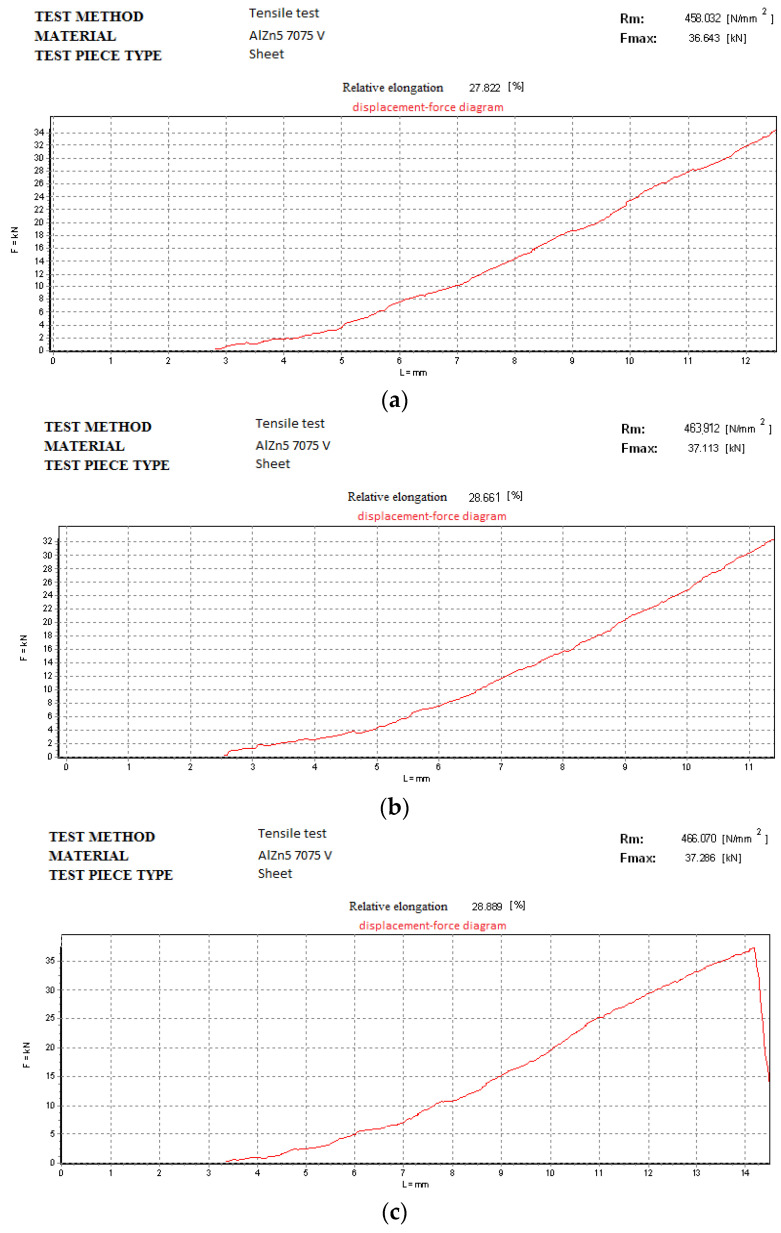
Tensile test for the specimens welded with vibrations: (**a**) test piece 1; (**b**) test piece 2; (**c**) test piece 3 from Figure 10b.

For the test pieces welded without vibrations (Figure 11) the authors obtained the following values: (a) 409.225 N/mm^2^ for test piece 1; (b) 401.378 N/mm^2^ for test piece 2; (c) 404.125 N/mm^2^ for test piece 3. Figure 12 depicts the graphs for the test specimens welded on a vibrating surface. In both cases the test specimen broke at similar forces for their specific procedure.

For the test pieces welded with vibrations (Figure 12) the authors obtained the following values: (a) 458.032 N/mm^2^ for test piece 1; (b) 463.912 N/mm^2^ for test piece 2; (c) 466.070 N/mm^2^ for test piece 3.

### 3.5. Energy-Dispersive X-Ray Spectroscopy

**Figure 13:**
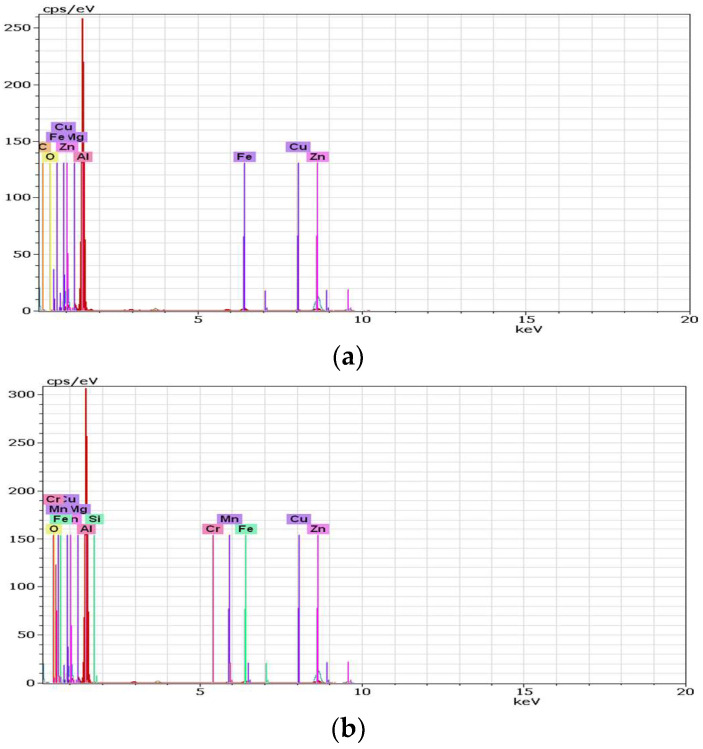
EDX spectrum of the MIG welding without longitudinal vibrations for (**a**) test piece 1; (**b**) test pieces 2 and 3.

**Figure 14:**
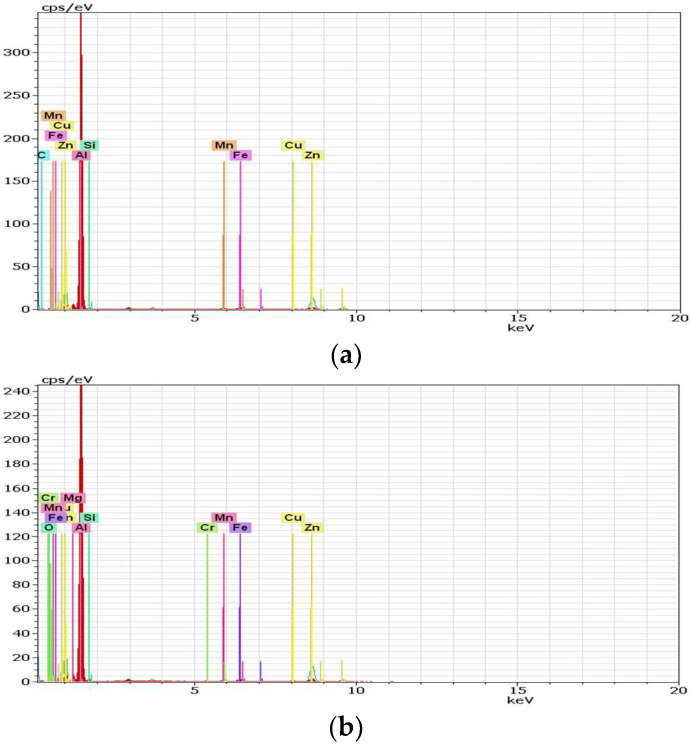
EDX spectrum of the MIG welding with longitudinal vibrations for (**a**) test piece 1; (**b**) test pieces 2 and 3.

Energy-Dispersive X-ray Spectroscopy (EDX, TESCAN, Brno, Czech Republic) analysis can provide the chemical characterization of each specimen, as seen in Figure 13 and Figure 14. It can also highlight precipitation phenomena, which is the reason for applying this method.

## 4. Conclusions

This paper presents a comprehensive experimental study that aims to characterize and analyze the microstructural and mechanical properties of an Al 7075 alloy welded with MIG both without and with forced longitudinal vibrations, using the Al 4043 alloy as filler material. The use of forced longitudinal vibrations represents a heat treatment that is applied during welding, which does not require other post-welding treatments.

As a consequence of using Al 4043 as filler material, the HAZ becomes strengthened through precipitation during welding, especially due to additions of Mg and Cu. By introducing forced longitudinal vibrations in to the weld, the risk of hot cracking can be mitigated, and thus we obtain a lower level of hydrogen inclusions in a higher-density grain microstructure (fragile Mg_2_Si grains). This results from the chemical composition concentrations, elemental mapping and EDX analysis provided in this study.

The microscopic observations, consisting in applying SEM on the HAZ and cross-section of the welds, have shown that the welds have a band structure, especially after the MIG processes are performed. Although welding of aluminum alloys encounters difficulties in regard to the removal of aluminum oxide, when mechanical vibrations are conducted as described in this study and highlighted by using SEM, an acceptable structure free of the characteristic porosity can be obtained.

The determinations for hardness performed in this study reveal an increase in Vickers HV on the cross-sections of the weld, performed under vibrations, by 8.7% and an increase of 12.5% in the HAZ of the same weld under vibrations compared to no vibrations being applied.

According to the data obtained for the tensile tests performed within this study, the authors notice an increase of 12–15.5% in tensile strength for the aluminum plates welded under longitudinal vibrations compared to no vibrations being applied.

Through grain structure growth and formation, it is possible to identify how alloy properties are influenced during welding. By controlling the nucleation and solidification process to a certain extent when welding, it is possible to modify the microstructural characteristics and consequently the mechanical properties of the weld. By technological optimization of the manner in which the metal pool is solidified (crystallizes), the authors show that alloys with low weldability can offer a specific set of properties that can prove to be appropriate. By inducing forced longitudinal vibrations, transmitted along the formation of the metal bed during welding, dissolved gasses are released and the grain density increases. The changes obtained are due to the influence of the frequency level, which alters the structure both in the fusion zone and in the middle of the weld, thus increasing the hardness. The aim of this research was to emphasize the differences between the effects of vibrations in MIG-welded Al 7075 (AlZn5.5MgCu) alloy specimens and the classic MIG welding procedure (without vibrations).

## Figures and Tables

**Table 1:** Technical welding characteristics.

Specimen	I [A]	U [V]	f [Hz]	S [cm/min]	Sfw [m/min]	Q Argon [L/min]
Al 7075, vibrated	192	20	50	45	8.1	15
Al 7075, stationary	192	20	–	45	8.1	15

**Table 2:** Chemical composition of the Al 7075 alloy (AlZn5.5MgCu) welded in the experiment.

Alloy Designation	Si	Fe	Cu	Mn	Mg	Cr	Zn	Ti	Others (%)
EN AW-7075 *	Max. 0.4	Max. 0.5	1.2–2	Max. 0.3	2.1–2.9	0.18–0.28	5.1–6.1	Max. 0.2	Max. 0.15

* According to SR EN 573-3 [71].

**Table 3:** Chemical composition of the Al 4043 alloy (AlSi5) used as filler material.

Alloy Designation	Si	Fe	Cu	Mn	Mg	Zn	Ti	Ga	Others (%)
EN AW-4043A *	4.5–6	Max. 0.8	Max. 0.3	Max. 0.05	Max. 0.05	Max. 0.1	Max. 0.2	-	0.05

* According to SR EN 573-3 [71].

**Table 4:** Longitudinal shear strength comparison of aluminum filler alloys.

Filler Alloy	Longitudinal Shear Strength (MPa)
4043	80
4047	80–90
4643	93
5183	127.6
5356	117.2
5554	103.4

**Table 5:** Chemical composition (%) of the Al 7075 alloy (AlZn5.5MgCu) determined experimentally without vibrations.

Alloy Designation	Si	Fe	Cu	Mn	Mg	Cr	Zn	Ti
EN AW-7075 *	1.6	0.5	0.92	0.2	2	0.15	3.5	0.18

* MIG welded without vibrations.

**Table 6:** Chemical composition (%) of the Al 7075 alloy (AlZn5.5MgCu) determined experimentally with vibrations.

Alloy Designation	Si	Fe	Cu	Mn	Mg	Cr	Zn	Ti
EN AW-7075 *	2	0.6	1	0.22	2.5	0.18	3	0.16

* MIG welded with vibrations.

**Table 7:** Microhardness in the HAZ of the welded specimens without longitudinal vibrations.

Place of Indentation	HV100 gf	Imprint Size Diag.x	Imprint Size Diag.y
HAZ	97.8	0.0467 µm	0.0404 µm

**Table 8:** Microhardness in the weld without longitudinal vibrations.

Place of Indentation	HV100 gf	Imprint Size Diag.x	Imprint Size Diag.y
Weld	75.8	0.0511 µm	0.0484 µm

**Table 9:** Microhardness in the HAZ of the welded specimens with forced longitudinal vibrations.

Place of Indentation	HV100 gf	Imprint Size Diag.x	Imprint Size Diag.y
HAZ	110	0.0447 µm	0.0376 µm

**Table 10:** Microhardness in the weld with forced longitudinal vibrations.

Place of Indentation	HV100 gf	Imprint Size Diag.x	Imprint Size Diag.y
Weld	82.4	0.0527 µm	0.0422 µm

## Data Availability

The original contributions presented in this study are included in the article. Further inquiries can be directed to the corresponding author.
